# The Influence of Epilepsy on Oral Health Outcomes: A Retrospective Study in South Indian Adults

**DOI:** 10.7759/cureus.66101

**Published:** 2024-08-03

**Authors:** Sowmya S, Haripriya A

**Affiliations:** 1 Oral Medicine, Radiology and Special Care Dentistry, Saveetha Dental College and Hospitals, Saveetha Institute of Medical and Technical Sciences, Saveetha University, Chennai, IND

**Keywords:** oral prophylaxis, antiepileptic medications, special care patients, oral health, epilepsy disorders

## Abstract

Introduction

Epilepsy, a chronic neurological disorder marked by recurrent seizures, affects approximately 50 million people worldwide with a higher prevalence in developing countries. This condition challenges motor skills and coordination, leading to poor oral health maintenance. The study aimed to evaluate the effect of epilepsy on oral health outcomes in adults by contrasting South Indian epileptics with healthy controls. The primary objective was to assess the prevalence of oral health issues in patients with epilepsy compared to healthy individuals and to analyze the types and frequency of dental procedures required in epileptic patients compared to healthy controls in the South Indian population.

Materials and methods

A retrospective study was conducted in the Department of Oral Medicine and Radiology, Saveetha Dental College and Hospitals, Chennai, India. Approved by the Institutional Human Ethical Committee (Registration ID: IHEC/SDC/OMED-2202/23/106), the study involved 105 epileptic patients and 105 healthy controls from records between January 2021 and December 2023. Both male and female patients within the age limit of 18-55 years were included. Statistical analysis was performed using IBM SPSS Statistics for Windows, Version 29.0 (Released 2022; IBM Corp., Armonk, New York, United States).

Results

The study involved 210 participants with an equal gender distribution. Valproate was the most common medication used by 39% of epileptic patients. Gingival hyperplasia was significantly more prevalent in the epileptic group (24%). The epileptic group also required more dental procedures, with 32% of teeth needing restoration, 20% root canal treatment, and 20% extraction, compared to 12%, 11%, and 5%, respectively, in the control group.

Conclusion

Epileptic patients exhibit poorer oral health outcomes, including higher rates of gingival hyperplasia and a greater need for dental procedures compared to healthy controls. These findings highlight the necessity for targeted dental care and regular monitoring for individuals with epilepsy to improve their oral health and overall quality of life.

## Introduction

Epilepsy is a chronic neurological disorder marked by recurrent seizures, which are brief episodes of uncontrolled electrical activity in the brain. These seizures cause sudden changes in body movement, sensations, emotions, or consciousness, ranging from mild twitching to full-body convulsions that can sometimes affect bowel or bladder control [1]. According to the World Health Organization (WHO), epilepsy affects approximately 50 million people globally, highlighting its status as a significant public health concern. This prevalence is not uniformly distributed across the world; in developing nations, the incidence of epilepsy is significantly higher, with about 43 out of every 1,000 individuals affected, compared to lower rates observed in more developed countries. This disparity may be attributed to various factors including limited access to healthcare, higher rates of infections, and other socioeconomic determinants [2,3]. Oral health in individuals with epilepsy is often compromised due to challenges with motor skills and coordination. These difficulties lead to a higher prevalence of tooth decay and missing teeth than the general population [4]. The motor impairments associated with epilepsy, including issues with fine and gross motor control, make it challenging for patients to perform tasks such as effective tooth brushing [5,6]. This complex interplay between epilepsy and dental care necessitates further investigation to guide optimal treatment strategies for these patients [7]. Despite the recognized needs of patients with epilepsy, there is a significant gap in dental literature regarding the effective management of their oral health [8]. The primary goal of epilepsy treatment is to improve the quality of life by controlling seizures and minimizing the adverse effects of medications. Epilepsy can significantly impact oral health, particularly in individuals experiencing poorly controlled tonic-clonic seizures. These seizures, characterized by uncontrolled muscle contractions, often result in minor oral injuries like tongue biting, as well as more serious issues such as tooth fractures, displacements, or facial trauma [9]. The interplay between epilepsy and dental care requires further investigation to develop optimal treatment strategies tailored to this population. Despite the recognized dental health needs of patients with epilepsy, there remains a notable gap in the dental literature concerning effective management practices for their oral health. Addressing this gap is essential to improving the quality of life for individuals with epilepsy by controlling seizures and minimizing the adverse effects of antiepileptic medications. To manage and prevent oral complications effectively, dentists need a comprehensive understanding of epilepsy and its oral health implications. With this knowledge, they can tailor their treatment approaches, adapt techniques for motor skill-limiting patients, and collaborate with neurologists to ensure optimal care. This holistic approach promotes oral health and safety for individuals with epilepsy [4]. Early management and prevention through regular dental health monitoring are essential for the overall well-being of individuals with epilepsy [10]. Therefore, the study aims to assess the oral health outcomes in epileptic patients compared to healthy controls, providing crucial insights into this population's specific dental care needs.

## Materials and methods

A retrospective study was performed in the Department of Oral Medicine and Radiology at Saveetha Dental College and Hospitals in Chennai, India, to assess the oral health outcomes in epileptic adults compared to healthy controls in the South Indian population. The study received approval from the Institutional Human Ethical Committee of Saveetha Dental College (Registration ID: IHEC/SDC/OMED-2202/23/106). The researchers obtained 105 case records of patients diagnosed with epilepsy and 105 records of healthy adults without systemic illness from an electronic database from January 2021 to December 2023. A convenience sampling technique was used in the present study. Both male and female patients aged 18-70 years were included in the study.

Training and calibration

Two researchers underwent a two-month training and calibration period for patient selection in this study. The training involved a comprehensive review of the eligibility criteria and meticulous patient assessment. During the calibration process, the researchers practiced patient selection under the guidance of experienced colleagues to ensure consistency and accuracy.

Sample size calculation 

In this study, a simple randomized sampling method was utilized. The sample size was determined using G*Power, a statistical software widely employed for calculating sample sizes in research. This method, informed by supporting literature on a similar topic [8], ensured that the sample size was sufficient to meet the study's objectives and maintain statistical power.

Inclusion and exclusion criteria

In this study, we focused on comparing the oral health of patients with a history of epilepsy to that of healthy controls with no systemic diseases. The participants included both males and females from the South Chennai zone. Specific inclusion and exclusion criteria were established to ensure a homogenous sample and reliable results. Inclusion criteria encompassed patients with a documented history of epilepsy under consistent medical supervision and healthy control patients with no systemic diseases, providing a baseline for comparison. Both male and female patients within the 18-70 age range, with an average age of 50, were included. Exclusion criteria ruled out epilepsy patients not under medical supervision, pregnant patients due to the unique effects of pregnancy on oral health, individuals under 18 years old because of differing oral health baselines and developmental factors, and patients with other systemic illnesses such as hypertension, diabetes, and coronary artery diseases to avoid confounding variables. Additionally, patients with a history of malignancies undergoing chemotherapy or radiotherapy, which significantly impact oral health, were excluded, as well as those with any other comorbidities that could influence oral health, to maintain the study's focus on the effects of epilepsy alone. By implementing these criteria, the study aimed to isolate the impact of epilepsy on oral health, ensuring that the comparisons between the epileptic and healthy control groups were as accurate and meaningful as possible.

Statistical analysis

The collected data were imported into Microsoft Excel 2016 (Microsoft Corporation, Redmond, Washington, United States) and analyzed using IBM SPSS Statistics for Windows, Version 29.0 (Released 2022; IBM Corp., Armonk, New York, United States). Descriptive statistics, including frequency and percentage analyses, were employed to interpret the data.

## Results

**Figure 1:**
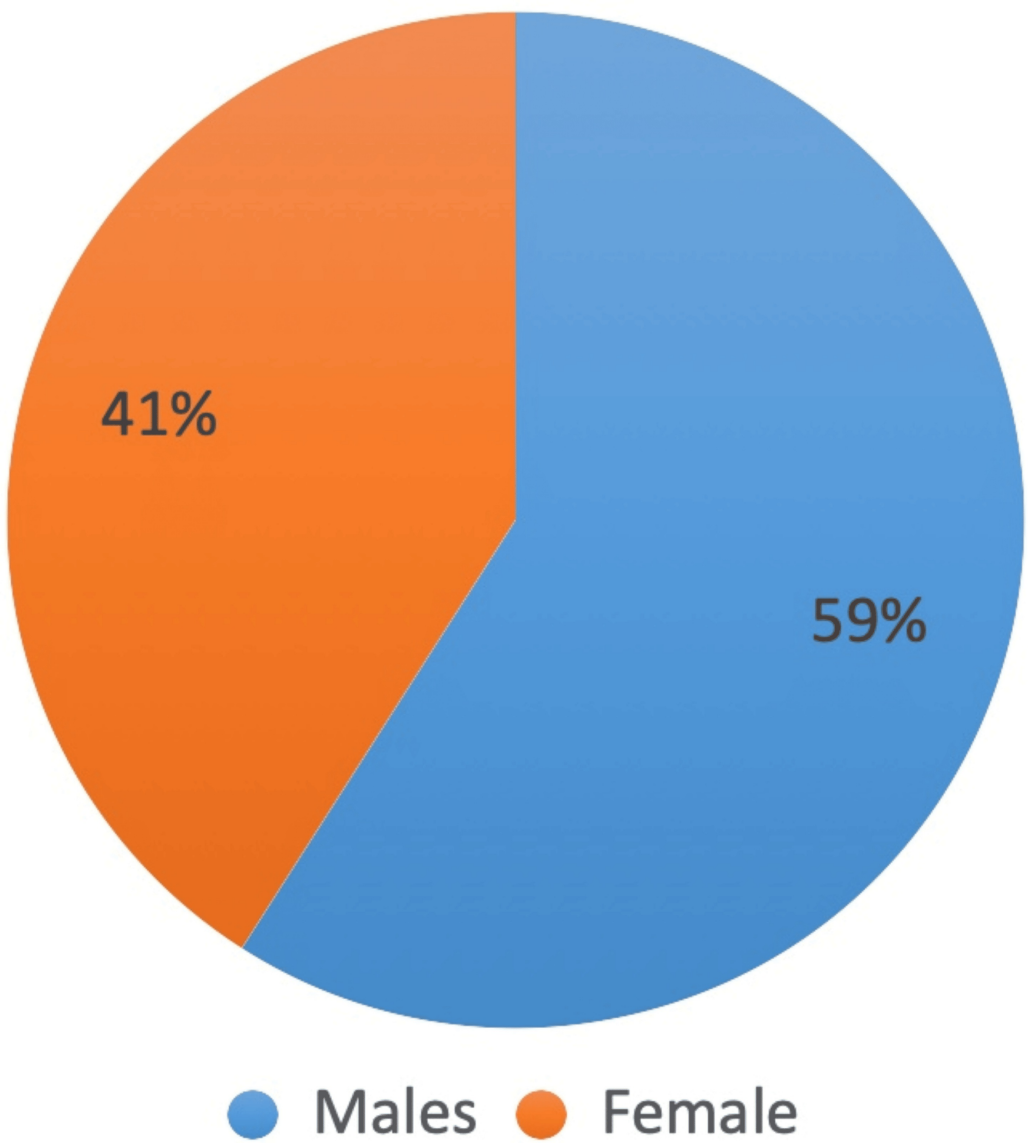
Gender distribution in epileptic and control groups.

The study involved 210 participants, with an equal distribution of males and females across both the epileptic and control groups. Specifically, there were 58 males and 47 females in each group. This balanced gender distribution allows for a more accurate comparison of oral health outcomes between individuals with epilepsy and healthy controls. By ensuring that each group had the same number of male and female participants, the study aimed to eliminate gender as a confounding factor, thus providing more reliable and valid results regarding the impact of epilepsy on oral health (Figure 1).

**Figure 2:**
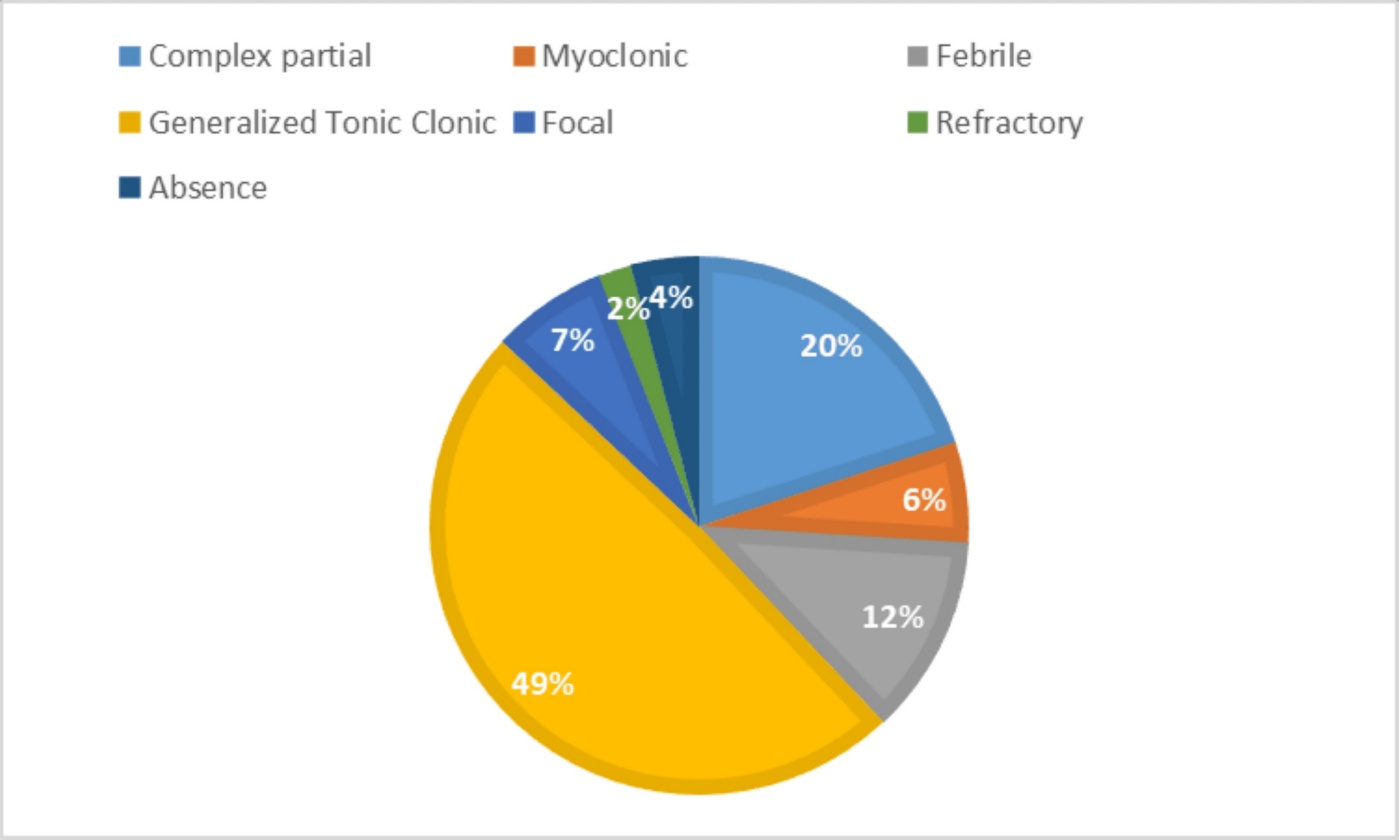
Types of epilepsy observed within the epileptic group.

The epileptic group included patients with various types of epilepsy and associated medications. Generalized tonic-clonic seizures were present in 49% of the patients, complex partial seizures in 20%, febrile seizures in 12%, focal seizures in 7%, myoclonic seizures in 6%, absence seizures in 4%, and refractory seizures in 2% (Figure 2).

**Figure 3:**
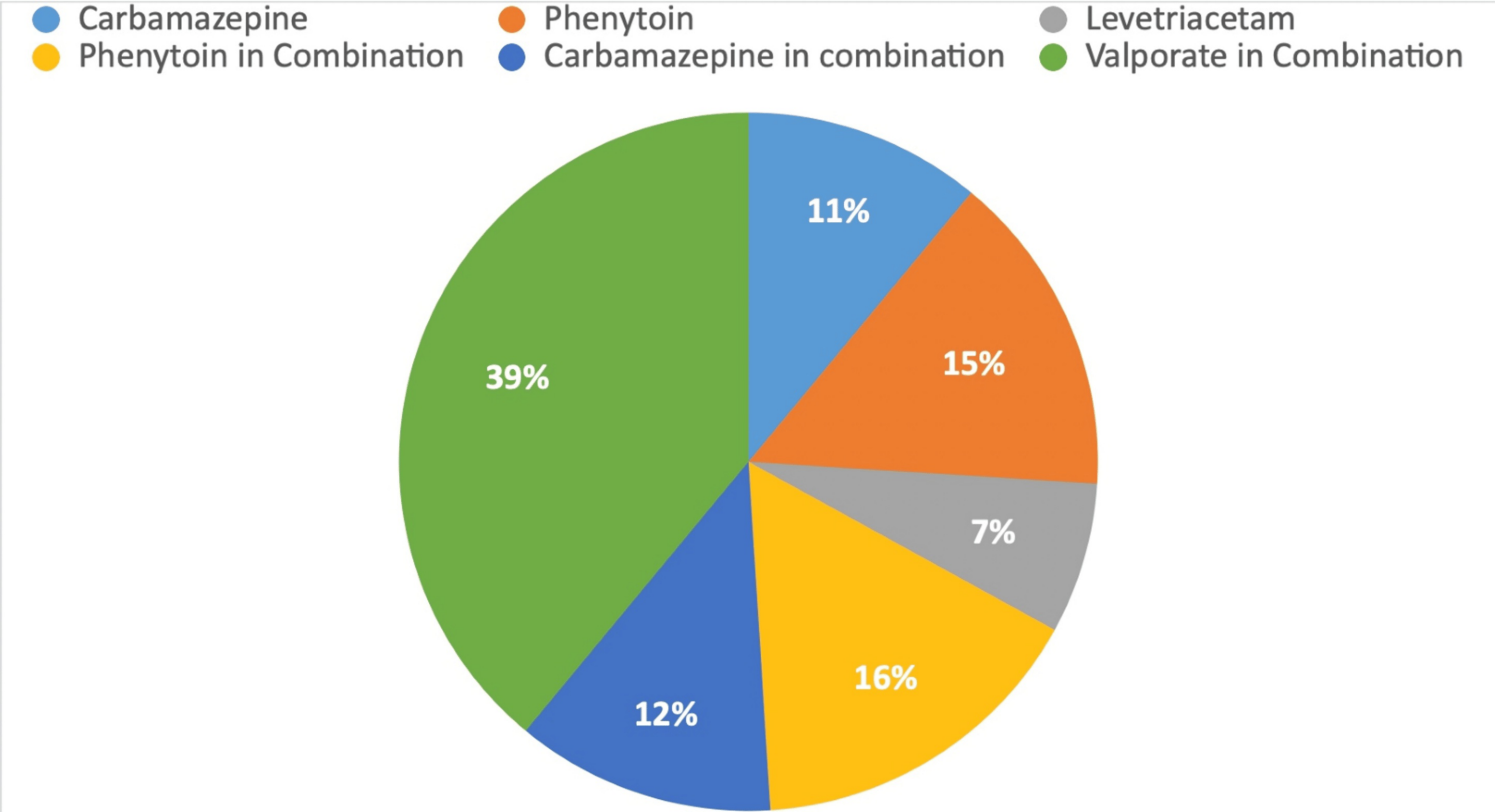
Types of medication taken by epileptic patients.

Valproate, frequently used in combination with other medications, was the most commonly prescribed drug, administered to 39% of the patients. Phenytoin was used by 16% of the patients in combination with other drugs, while 15% were on phenytoin alone. Carbamazepine was prescribed to 12% of the patients in combination with other medications, and 11% were on carbamazepine alone. Additionally, 7% of the patients were treated with levetiracetam (Figure 3).

**Figure 4:**
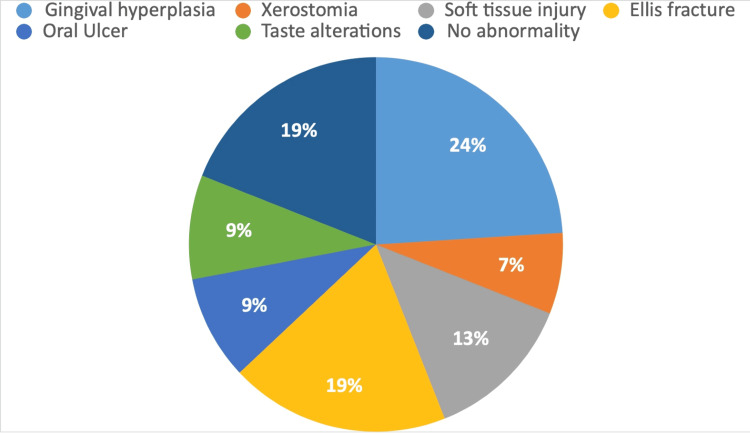
Oral manifestations observed in the epileptic group.

Gingival hyperplasia (gum overgrowth) was significantly more common in the epileptic group, affecting 24% of the patients. Additionally, 19% of the patients experienced Ellis fractures, which are various types of tooth fractures. Soft tissue injuries, such as damage to the lips, cheeks, tongue, and other oral tissues, were observed in 13% of the patients. Taste alterations and the presence of oral ulcers were reported by 9% of the patients. Notably, 19% of the patients showed no oral abnormalities, suggesting that a substantial portion of the epileptic group maintained good oral health (Figure 4).

**Figure 5:**
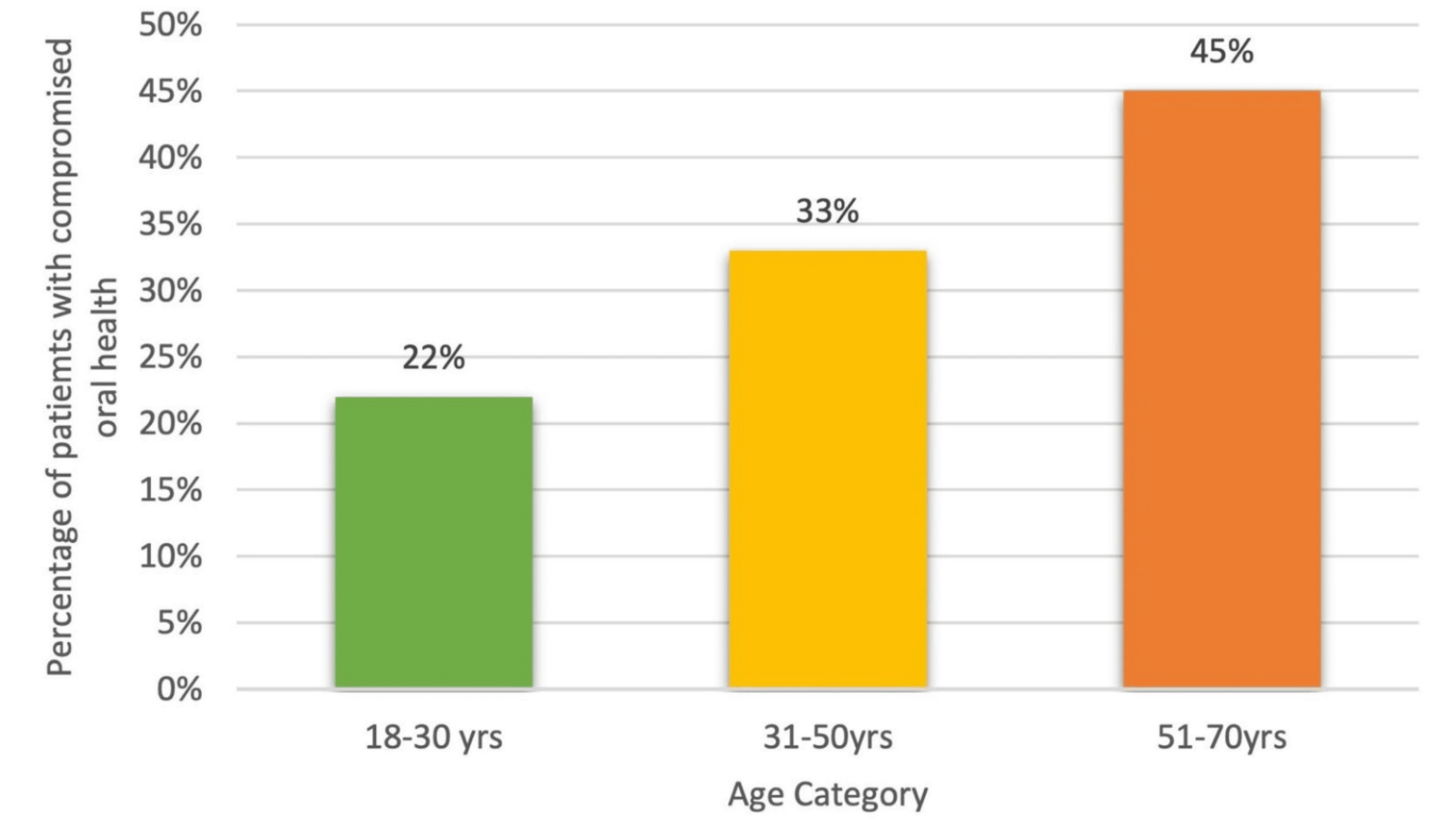
Percentage of patients with compromised oral health across different age categories.

The graph demonstrates an increasing trend in the percentage of patients with compromised oral health across different age groups. Younger adults (18-30 years) exhibit the lowest rate at 22%, middle-aged adults (31-50 years) show a higher rate at 34%, and older adults (51-70 years) have the highest rate at 45%. This pattern suggests a correlation between aging and a greater likelihood of compromised oral health, highlighting the need for enhanced oral healthcare and monitoring as individuals age (Figure 5).

**Figure 6:**
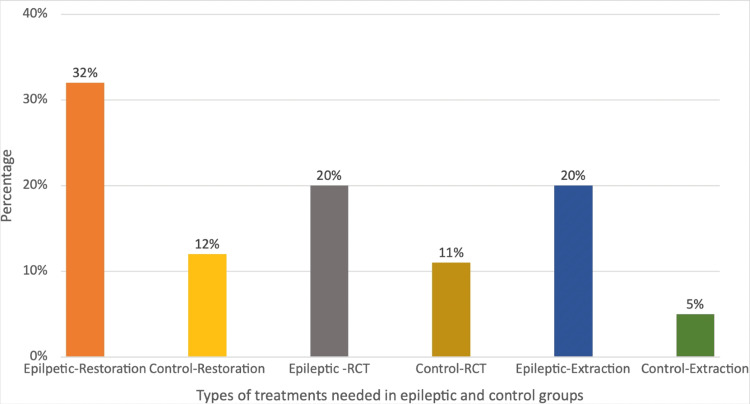
Percentage of teeth that needed restoration, RCT, and extraction. RCT: root canal treatment

Moreover, the epileptic group required more dental procedures than the control group. In the epileptic group, 32% of teeth needed restoration, 20% required root canal treatment, and 20% needed extraction. In contrast, in the control group, only 12% of teeth required restoration, while 11% and 5% needed root canal treatment and extraction, respectively (Figure 6).

## Discussion

Patients with special healthcare needs often neglect their dental hygiene, leading to deteriorating oral health [11]. Those with conditions like epilepsy face additional challenges, experiencing higher rates of missing and decayed teeth, fewer restored or replaced teeth, and potential adverse effects from antiepileptic medications [12]. While most patients with well-controlled seizures can receive outpatient dental care, dentists must be knowledgeable about epilepsy and its medications. A thorough medical history is essential for all dental patients, especially those with epilepsy, who often have other medical and psychiatric conditions. Patients should ideally consult their neurologists to ensure personalized care. Collaboration between neurologists and dentists is the key, involving discussions about seizure types, frequency, severity, and circadian periodicity to schedule dental visits when the risk of seizures is lower [13]. McHugh and Delanty [14] in the year 2008 showed that in worldwide gender determination, females have a marginally smaller annual incidence of epilepsy than males analogous to the current study, showcasing that the incidence of epilepsy was higher in males by 59% compared to females. Research suggests that men are slightly more likely than women to experience epilepsy. For instance, out of every 1,000 people, 7.31 men and 6.85 women suffer from the condition. This discrepancy may be due to various factors, including men's greater susceptibility to risk factors such as head injuries, which are more common in this population due to their participation in high-risk occupations and activities, as well as deleterious habits and frequent trauma [15].

The study conducted by Kammerman and Wasserman indicated that tonic-clonic (grand-mal) seizures are the most prevalent type of generalized seizure in adults, occurring in 25% of all patients with seizures [16], correlating with our study where 49% of epileptic patients presented with generalized tonic-clonic seizures. In 2023, a study carried out by Goswami et al. indicated that gingival hyperplasia develops in 50% of patients using antiepileptic drugs within 12-24 months of initial treatment. Epileptic seizures frequently result in minor oral injuries, such as tongue biting, and can also cause tooth damage, maxillofacial trauma, and, in some instances, tooth fractures [4]. We have noted that 24% of patients experienced gingival hyperplasia, 19% had Ellis fractures, 13% sustained soft tissue injuries, 9% had oral ulcers, 7% experienced xerostomia (dry mouth), and 19% had no abnormalities. As gingival hyperplasia is most commonly encountered in epileptic patients, dental treatment planning for medically induced gingival hyperplasia focuses on preventing and minimizing the condition. In cases of moderate to severe overgrowth, surgical reduction or laser may be necessary, but recurrence is possible if the medications causing the issue are still in use. Periodic surgical interventions may be needed, emphasizing the importance of the dentist's role in reinforcing oral hygiene and maintenance care. Additional strategies include collaborating with the neurologist to review and adjust medications, emphasizing meticulous oral hygiene, applying topical antimicrobial agents, utilizing laser therapy, and educating patients about the importance of consistent oral care and regular dental visits. By employing these measures, dentists can effectively manage and treat gingival hyperplasia, improving oral health outcomes. In an epidemiological investigation, Károlyházy et al. showed that patients with epilepsy exhibited notably poor oral hygiene compared to their healthy counterparts. The authors suggest that the elevated incidence of dental issues, such as caries, injuries, and periodontal disease, resulted from a combination of factors, including inadequate oral hygiene practices, limited socioeconomic resources, and oral cavity injuries [17]. Our study indicates that the number of teeth that required restoration, root canal treatment, and extraction in epilepsy patients was 32%, 20%, and 20%, whereas only 12%, 11%, and 5% of teeth required these treatments in the control group, respectively. The complexities surrounding oral health factors are significant, particularly for individuals with epilepsy in India, where ignorance and stigma towards epilepsy exacerbate their condition, making it more challenging than the disease itself.

When considering evidence from various studies and the potential obstacles to oral healthcare faced by epileptic individuals, it is reasonable to conclude that their oral health is inferior to that of the general population. Epileptic patients require special attention to their oral hygiene and dental care to improve their overall well-being and quality of life while minimizing the need for invasive treatments. They often face motor skill coordination challenges [17], making it difficult to maintain optimal oral hygiene with manual toothbrushes; electric toothbrushes can help by requiring less manual dexterity and consistent brushing motion. Educating guardians or caregivers about the importance of oral health is crucial, including teaching proper brushing and flossing techniques and encouraging regular checks [18]. Oral health education tailored to different age groups can benefit individuals with epilepsy. For young adults, using adaptive oral hygiene tools such as electric toothbrushes can ease the challenges posed by motor skill difficulties. Regular dental visits are crucial, especially if seizure frequency or medication changes impact oral health. Additionally, managing stress through exercise, relaxation techniques, and adequate sleep is essential to prevent conditions like bruxism (teeth grinding). Middle-aged adults with epilepsy should pay special attention to bone health, as long-term use of antiepileptic drugs can affect bone density. Incorporating calcium and vitamin D supplements into their diet can help mitigate this risk. Managing dry mouth, a common side effect of medication, is also important; using saliva substitutes or stimulants and staying well-hydrated can prevent oral discomfort and decay. For older adults, proper care of dental appliances such as dentures is essential. Ensuring these appliances fit well is crucial to avoid complications during seizures. Regular check-ups with a dentist can help maintain the proper fit and function of these appliances, ensuring overall oral health and comfort.

Dentists play a significant role by collecting a detailed history of seizures, medications, and any auras and communicating with the patient's neurologist for pre-treatment planning [19]. Key considerations include preparing a seizure management plan, ensuring a calm environment, having emergency protocols in place, scheduling appointments when seizures are least likely, considering sedation for severe anxiety, and promoting preventive care with more frequent check-ups and fluoride treatments. Regular dental check-ups every three months are crucial, along with preventive measures like tongue-biting devices and splints to protect the teeth during frequent seizures [20,21]. All dental treatments should be coordinated with the patient's neurologist to ensure safety and effectiveness [21,22]. This collaborative approach helps maintain oral hygiene, allows for early diagnosis, and provides shorter treatment times, ultimately promoting optimal oral health for epileptic patients.

The study's limitations include its single-center design which may limit the generalizability of the findings to other populations with different demographic and clinical profiles. Conducting prospective studies with larger sample sizes and longer follow-up periods would provide more comprehensive and reliable evidence regarding oral health outcomes in individuals with epilepsy. Future research should incorporate longitudinal and multicenter studies to provide a comprehensive understanding of the long-term impact of oral health interventions on individuals with epilepsy. Such studies would enable researchers to track changes over extended periods and across diverse populations, offering more robust and generalizable findings. Additionally, there is a need to explore the correlation between systemic diseases and epilepsy in the pediatric population. Investigating how systemic conditions influence epilepsy in children, and vice versa, could uncover important insights into the management and treatment of these patients, ultimately improving their overall health outcomes.

## Conclusions

This retrospective study conducted among the South Indian population revealed that adults with epilepsy had significantly poorer oral health compared to healthy controls. Epileptic patients exhibited a higher prevalence of gingival hyperplasia, likely exacerbated by anticonvulsant medications, and required more extensive restorative procedures, including fillings, crowns, root canals, and extractions. The increased need for these treatments highlights the substantial impact of epilepsy on oral health, necessitating tailored dental care approaches for this population. The findings underscore the critical importance of integrating special care dentistry into the management of individuals with epilepsy. Regular dental check-ups, meticulous oral hygiene maintenance, and preventive measures should be prioritized to mitigate the adverse effects of epilepsy and its treatment on oral health. Multidisciplinary collaboration between dental professionals, neurologists, and caregivers is essential to address the unique oral health challenges faced by epileptic patients, ensuring comprehensive and effective care. By emphasizing the necessity of specialized dental care, this study advocates for heightened awareness and proactive strategies to improve oral health outcomes in individuals with epilepsy. Enhanced education on oral hygiene practices, timely dental interventions, and personalized treatment plans can significantly contribute to better overall health and quality of life for this vulnerable population.
